# The effect of microstructure on mechanical and magnetic properties of FeCoNiAl_0.75_Nb_0.25_ high-entropy alloy†

**DOI:** 10.1039/d5ra00358j

**Published:** 2025-03-06

**Authors:** Minh Duc Le, Thanh Hung Nguyen, Van Duong Nguyen, Mai Khanh Pham, Hong Hai Nguyen

**Affiliations:** a School of Materials Science and Engineering, Hanoi University of Science and Technology Hanoi Vietnam hai.nguyenhong@hust.edu.vn; b Faculty of Mechanical Engineering, Le Quy Don Technical University Hanoi Vietnam

## Abstract

This work systematically investigated the relationship between the microstructure, and mechanical and magnetic properties of FeCoNiAl_0.75_Nb_0.25_ high-entropy alloy. Our results indicated that the microstructure of the alloy comprised a BCC solid solution phase along with a eutectic mixture of FCC and intermetallic phases. The application of heat treatment resulted in a significant evolution of the microstructure. The precipitation of the needle-like intermetallic phase occurred rapidly with increasing annealing temperature, reaching a maximum proportion at 825 °C, and decreased quickly upon further increase to 1000 °C. Correspondingly, the hardness and compressive yield strength of the alloy increased rapidly, attaining maximum values of approximately 600 HV and 2000 MPa, respectively. However, the precipitation adversely affected magnetic properties. The best values in the as-cast state for saturation magnetization, and coercive force are 0.67 T and 716 A m^−1^, respectively, while the hardness remains 493 HV. Therefore, it is very suitable for magnetic parts requiring superior mechanical properties.

## Abstract 2

This work systematically investigated the relationship between the microstructure, and mechanical and magnetic properties of FeCoNiAl_0.75_Nb_0.25_ high-entropy alloy.

## Graphical abstract

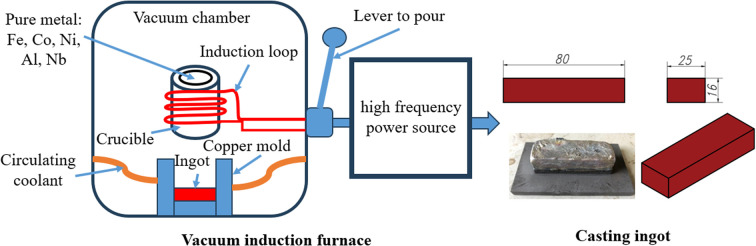

## 1. Introduction

High-entropy alloy (HEA) or multi-principal element alloy (MPEA) is a new alloy in materials science with many unique properties and broad application potential.^1^ HEA exhibits superior properties such as high hardness and wear resistance,^2^ the ability to maintain strength at high temperatures,^3,4^ good ductility at low temperatures^5,6^ and superplastic properties^7,8^ when changing composition and microstructure. The majority of published HEA compositions are based on transition metals such as Co, Cr, Fe, and Ni, and enriched with elements such as Al, Cu, Mn, V, Nb, Ti, and Mo.^1,9–21^ Nb is one of the alloying elements that can significantly influence the properties of FeCoNi-based high-entropy alloys. The microstructure of AlCoCrFeNb_*x*_Ni HEA consists of two phases: BCC solid solution phase and Laves phase (CoCr)Nb type. As the Nb content increases, the microstructure of the alloy transitions from hypoeutectic to hypereutectic. Correspondingly, both Vickers hardness and compressive yield strength exhibit a nearly linear increase.^22^ The AlCrFeNiCu alloy exhibits a two-phase structure with dendritic morphology. The addition of Nb facilitates the formation of fine eutectic structures, altering the grain morphology from cylindrical to equiaxed. The hardness, wear resistance, and compressive strength of the alloy improve with increased Nb content.^23^ Additionally, the yield strength of (CoCrFeMnNi)_100−*x*_Nb_*x*_ (*x* = 0, 4, 8, 12, 16) HEA increased quite rapidly from 202 to 1010 MPa. However, when increasing the Nb content, the fracture strain decreased very quickly from 60% to 12%.^24^ The microstructure of the CoCrFeNb_*x*_Ni (*x* > 0) HEAs changes significantly as *x* increases,^25^ from single-phase FCC solid solution structure to hypoeutectic, then to full eutectic and finally to a hypereutectic microstructure. As hardness and wear resistance increase, the plasticity decreases with increasing Nb content, due to the increase in the fraction of hard and brittle Laves phase. This shows the importance of Nb in the microstructure and properties of HEAs. Conversely, the current trend emphasizes reducing the number of elements and investigating new properties with high application potential, such as magnetic properties.^26^ Li *et al.*^27^ reported that increasing the molar fraction of Al to 0.7–1.0 in the FeCoNi-based alloys effectively reduces density while maintaining a relatively high saturation magnetization and low coercivity, consistent with a BCC structure. Additionally, the introduction of Nb enhances the stability of the BCC phase.^23^ Specifically, incorporating 0.25 molar fraction of Nb maintains low density while preventing excessive brittleness in the alloy.^23^ In this study, the FeCoNiAl_0.75_Nb_0.25_ alloy was designed to achieve a balance between reduced density and favorable magnetic properties. The relationship among the microstructure, mechanical properties, and magnetic properties of this alloy indicates its strong potential as a soft magnetic material for applications such as generator rotors and coil cores. Notably, it exhibits superior mechanical properties compared to currently used alloys.

## 2. Experimental procedures

**Fig. 1:**
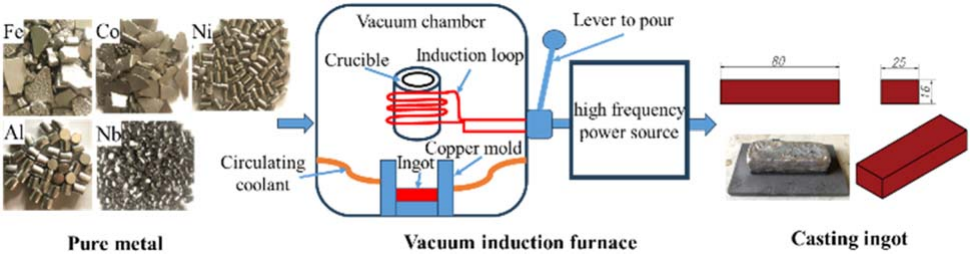
Schematic diagram of the fabrication process.

The FeCoNiAl_0.75_Nb_0.25_ alloy was synthesized using high-purity (>99.7%) elemental metals (Fe, Co, Ni, Al, Nb). The melting process was conducted in a vacuum induction furnace under a high-purity argon atmosphere, followed by casting into a water-cooled copper mold. The cast ingot has dimensions of 80 mm × 25 mm × 16 mm. Cut samples of size 5 mm × 5 mm × 5 mm were annealed at various temperatures of 600, 700, 825 and 1000 °C for 24 hours, followed by furnace cooling to room temperature. This prolonged annealing duration promotes the structural transformation from dendritic to equiaxed morphology, and facilitates the completion of the equilibrium precipitation. These samples denoted as HT-600, HT-700, HT-825 and HT-1000, respectively. Fig. 1 shows the fabrication process of the HEA samples. The XRD analysis was carried out using an Aeris diffractometer and Cu K_*α*_ radiation to determine the phase structure. The scanning speed is 3° min^−1^ with a scan range 20–80°.

The microstructure of the alloy was studied using an Axiovert A2M optical microscope, and secondary electron (SE) imaging in scanning electron microscopy (SEM) was utilized to analyze the surface morphology of the samples. The SEM images were acquired using a Jeol JSM-IT200 device at an accelerating voltage of 10 kV, with a working distance of 10 mm. The chemical composition of the ingot was analyzed using energy-dispersive spectroscopy (EDS), as listed in Table 1. It showing that the as-cast state composition is quite close to the nominal composition. For microstructural observation, specimens were ground to x2000 SiC paper, polished, and etched in aqua regia solution (HNO_3_ : HCl = 1 : 3). Vickers hardness (HV3) was measured on a Wilson Wolpert machine under a load of 3 kg for 15 s. Each sample was measured five times to get the average value. The magnetization curves were measured at room temperature using a Lake Shore 7404 vibrating sample magnetometer (VSM). The specimens were cut to dimensions of 5 mm in width, 2 mm in thickness, and 5 mm in length using a wire electrical discharge machine.

**Table 1:** Composition of the as-cast FeCoNiAl_0.75_Nb_0.25_ alloy (wt%)

Element	Fe	Co	Ni	Al	Nb
Nominal composition	25.74	27.17	27.05	9.33	10.71
Actual composition	26.98	27.44	26.56	7.27	11.75

## 3. Results and discussion

### 3.1 Phase analysis

**Fig. 2:**
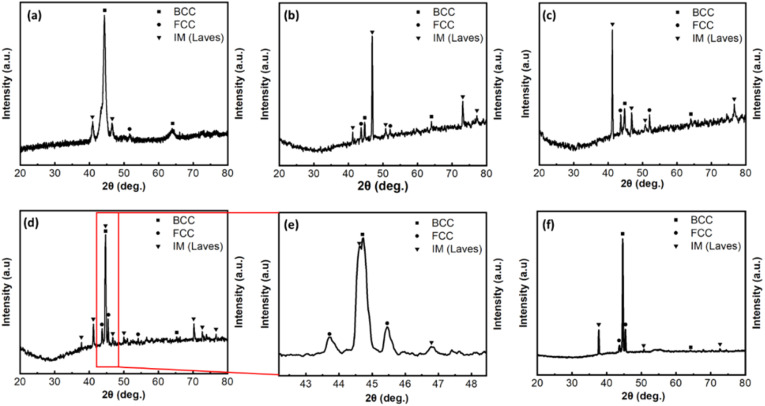
The XRD patterns of the alloy: (a) As-cast, (b) HT-600, (c) HT-700, (d) HT-825, (e) enlargement of 2*θ* = 42–48.5° region in HT-825 sample and (f) HT-1000.

In Fig. 2a, the XRD patterns reveal that the as-cast alloy consists of a mixture of FCC, BCC, and intermetallic (IM) phases. The BCC phase, with a lattice parameter of *a* = 0.2871 nm, is enriched in FeCoNiAl,^22^ while the FCC phase, characterized by a lattice parameter of *a* = 0.3592 nm, is predominantly FeCoNi-rich.^28^ The IM phase is identified as the (Nb_0.5_Al_0.5_)Co_2_ Laves phase, exhibiting a hexagonal close-packed (HCP) structure with lattice parameters *a* = 0.4752 nm and *c* = 0.7746 nm, as referenced in the PDF card. The diffraction pattern reveals that the BCC phase predominates, while the IM and FCC phases are present in smaller quantities. During heat treatment at 600 and 700 °C (Fig. 2b and c), the peak of the BCC phase is notably reduced, whereas the peaks of the FCC and IM phases are enhanced. When heat treatment is carried out at 825 °C, the IM phase reaches its peak, and the proportion of the BCC phase increases once more (Fig. 2d). In particular, at the BCC phase positions coexist two adjacent diffraction peaks. An additional peak corresponding to the IM phase appears as shown in Fig. 2e, this phase can be Co_2_Nb Laves phase with a cubic lattice structure according to PDF Card. The lattice parameters are *a* = 0.6759 nm. When the temperature reaches to 1000 °C, the proportion of the IM phase decreases clearly and mainly Co_2_Nb phase. We concluded that Al is depleted in the IM phase with increasing annealing temperature. The intensity of the FCC phase increases gradually with annealing temperature, then remains nearly constant. This is related with the high entropy effect that significantly lowers the Gibbs free energy of the system,^1^ which more easily yields the formation of solid solutions during solidification rather than ordered compounds, especially at the high temperature, and leads to the total number of phases well below the maximum equilibrium number allowed by the Gibbs phase rule.^1^

**Fig. 3:**
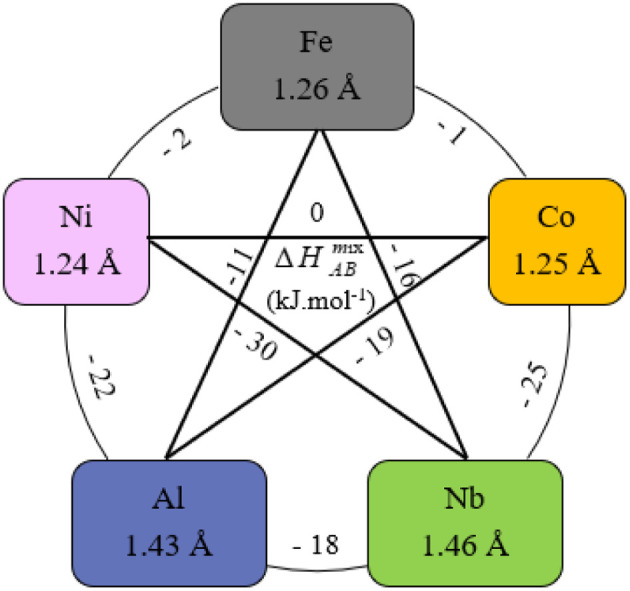
Values of Δ*H*^mix^_AB_,^31^ atomic radius of elements^32^ in FeCoNiAlNb HEA.

Several related research suggested that the formation of phases in high-entropy alloys can be predicted by thermodynamic parameters,^21,29,30^ including the difference in atomic size (*δ*), the mixing enthalpy (Δ*H*_mix_), the mixing entropy (Δ*S*_mix_), valence electron concentration (VEC), and *Ω* criterion:1
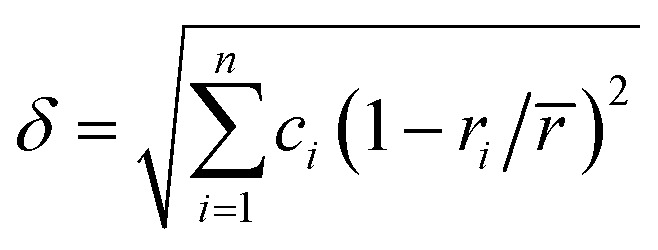
2
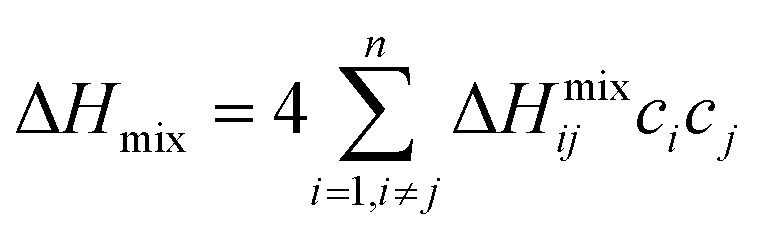
3
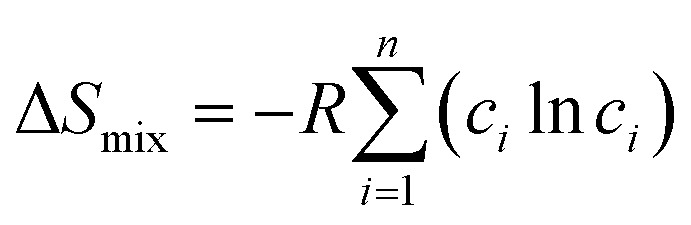
4
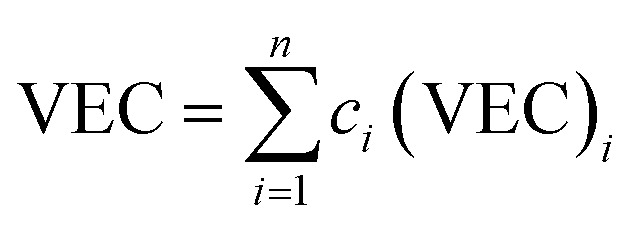
5
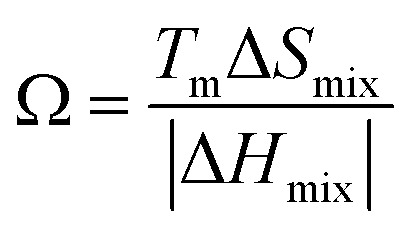
where 
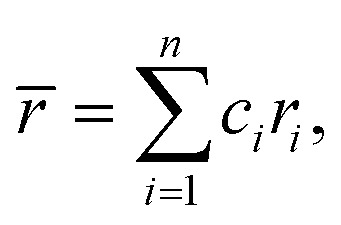
*r*_*i*_ is the Goldschmidt atomic radius of the *i*th element, as shown in Fig. 3. *c*_*i*_ is the molar ratio and Δ*H*^mix^_*ij*_ is the mixing enthalpy between the *i*th and *j*th elements, *T*_m_ is the average melting temperature, (VEC)_*i*_ valence electron concentration of element *i* and *R* = 8.314 J K^−1^ mol^−1^ is the gas constant.

According to the Hume–Ruthery rule, the difference in atomic size (*δ*) and the enthalpy of mixing (Δ*H*_mix_) are the two factors that solid solution phase stability in HEA. Guo *et al.*^32^ predicted that the easily formation of a solid solution phase corresponds to the region – 22 kJ mol^−1^ ≤ Δ*H*_mix_ ≤ 7 kJmol^−1^ and 0 < *δ* < 8.5%. On the other hand, M. Enoki et al.^33^ found that −20 kJ mol^−1^ ≤ Δ*H*_mix_ ≤ −10 kJ mol^−1^ can form additional IM phases in FeCoNi-based alloys. According to these two parameters, the alloy exhibits the values of – 15.78 kJ mol^−1^ and 6.3%, respectively, indicating a tendency to form a mixed phase including solid solution and IM phases.^21,33^ Moreover, the other parameters for forming a solid solution have been established: 11 ≤ Δ*S*_mix_ ≤ 19.5 (J K mol^−1^) and Ω ≥ 1.1.^29^ These precise conditions ensure that the solid solution phase is preferentially formed first, followed by the IM phase. Additionally, it should be noted that HEA with a VEC ≥ 8.0 tend to form an FCC phase, whereas a BCC phase is more likely to form when the VEC ≤ 6.87.^30^ Within the range of 6.87 ≤ VEC < 8.0,^30^ the FCC/BCC mixture phases are stable.

For the as-cast FeCoNiAl_0.75_Nb_0.25_ alloy, the values of these parameters after calculation are listed in Table 2. It can be seen that the values of parameters were consistent with the formation rules of solid solution. With the result from XRD pattern, the phase of this HEA mainly consists of the BCC, and a small portion of the FCC and IM phases.

**Table 2:** Thermodynamic parameters of the alloy

Δ*H*_mix_, kJ mol^−1^	Δ*S*_mix_, J K^−1^ mol^−1^	*δ*, %	VEC	*Ω*	Phase in this work
−15.78	12.7	6.30	7.63	1.35	FCC + BCC + IM

### 3.2 The as-cast microstructure

**Fig. 4:**
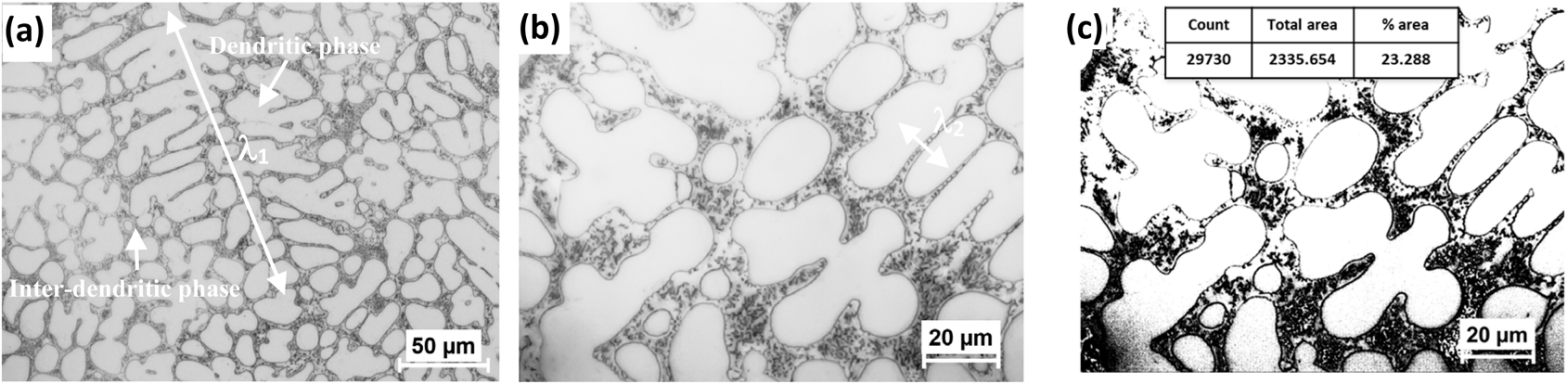
(a and b) The as-cast microstructure (optical microscope images), and (c) the fraction of interdendritic phase was determined using ImageJ software.

The as-cast microstructure of the FeCoNiAl_0.75_Nb_0.25_ alloy is illustrated in Fig. 4 and S1 of the ESI.† It can be seen that the microstructure is characterized by coarse dendritic phases measuring up to few hundred μm in length (*λ*_1_), as depicted in Fig. 4a. The distance between the secondary dendrites (*λ*_2_) is measured up to ∼11 μm, as shown in Fig. 4b. Additionally, the inter-dendritic phase is fine, the proportion of this phase is about 23%, as displayed in Fig. 4c.

**Fig. 5:**
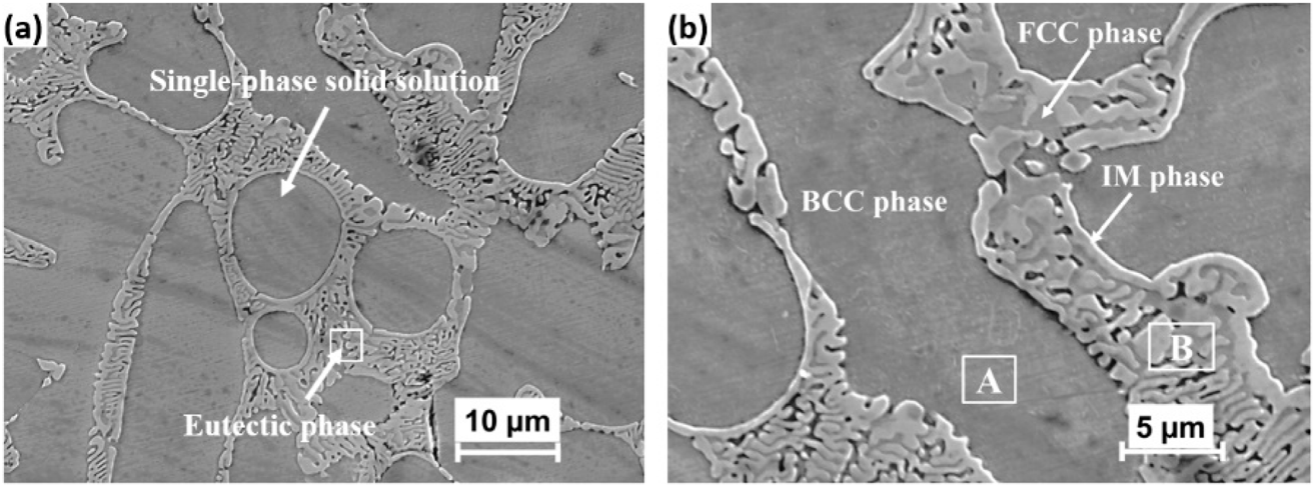
The secondary electrons SEM images of as-cast sample.

The SEM image of as-cast sample is depicted in Fig. 5. It reveals that the IM phase exhibits the morphology as a typical eutectic-like phase (Region B).^34^ While the dendritic region (Region A) is a single-phase solid solution. The EDS analysis results of the regions are shown in Table 3. It shows that solid solution phase (Region A) is enriched in Ni and Al and depleted in Nb and Fe. The nature of such observation corresponds to the BCC phase, as Al and Ni promote the formation of this phase.^22^ On the other hand, the eutectic phase (Region B) is rich in Fe, Co and Nb, but depleted in Al and Ni. The possible cause for this phenomenon is the relatively small atomic size of Ni (Fig. 3), which allows it to be readily dissolved into the supersaturated BCC solid solution during solidification, forming an interstitial solid solution. Moreover, Al and Ni have similar FCC lattice structures and considerable bonding energy due to their highly negative enthalpy of mixing (−22 kJ mol^−1^ for the Al–Ni atomic pair). Concerning Nb, it has the largest atomic size (0.146 nm) in this alloy system as well as very negative enthalpies of mixing with other alloying elements (Fig. 3), which are not favorable to form the solution with them, that is why Nb conversely enhances the tendency to segregate to form the ordered Nb-rich Laves phases.^22^ Based on the EDS analysis results in Table 4 and XRD pattern, the eutectic plates are rich in Co and Nb, suggesting they likely belong to the (AlNb)Co Laves phase. Meanwhile, the remaining phase of the eutectic structure is rich in Fe, Co, and Ni, but deplete in Al, corresponding to the FCC phase.^35^ Based on the above analysis, we can identify and label the phases, as described in Fig. 5b.

**Table 3:** The results of the EDS analysis (wt%)

	Fe	Co	Ni	Al	Nb
Region A (BCC phase)	24.33	27.38	33.33	10.65	4.29
Region B (eutectic phase)	29.00	28.00	17.64	3.64	21.71

**Table 4:** EDS analysis results of heat treatment samples^a^

Samples	Phase	Chemical composition (wt%)				
		Al	Fe	Co	Ni	Nb
HT-600	BCC phase	10.26	26.01	26.90	30.90	5.93
	Eutectic phase	2.90	23.39	25.82	20.55	27.33
	Eutectic plate (IM phase)	1.53	23.58	28.97	13.38	32.54
	Inter-eutectic plate (FCC phase)	3.98	30.33	28.26	32.32	5.11
HT-700	BCC phase	9.96	23.06	25.41	35.49	6.08
	Eutectic phase	3.85	24.75	26.66	24.73	20.02
	Eutectic plate (IM phase)	2.35	23.40	30.77	13.65	29.84
	Inter-eutectic plate (FCC phase)	3.83	30.43	29.49	31.16	5.09
HT-825	BCC phase	5.49	24.64	29.51	23.29	17.07
	Eutectic phase	0.56	20.13	29.51	15.59	34.11
	Precipitated phase	2.60	21.79	33.47	14.46	27.67
HT-1000	BCC phase	2.13	25.33	32.13	38.09	2.32
	Eutectic phase	0.92	23.44	31.04	13.01	31.58
	Precipitated phase	1.58	23.04	29.24	15.72	30.42

a An example of the EDS analysis position of the HT-700 sample is shown in Fig. S3.

### 3.3 The microstructure of annealed alloy

**Fig. 6:**
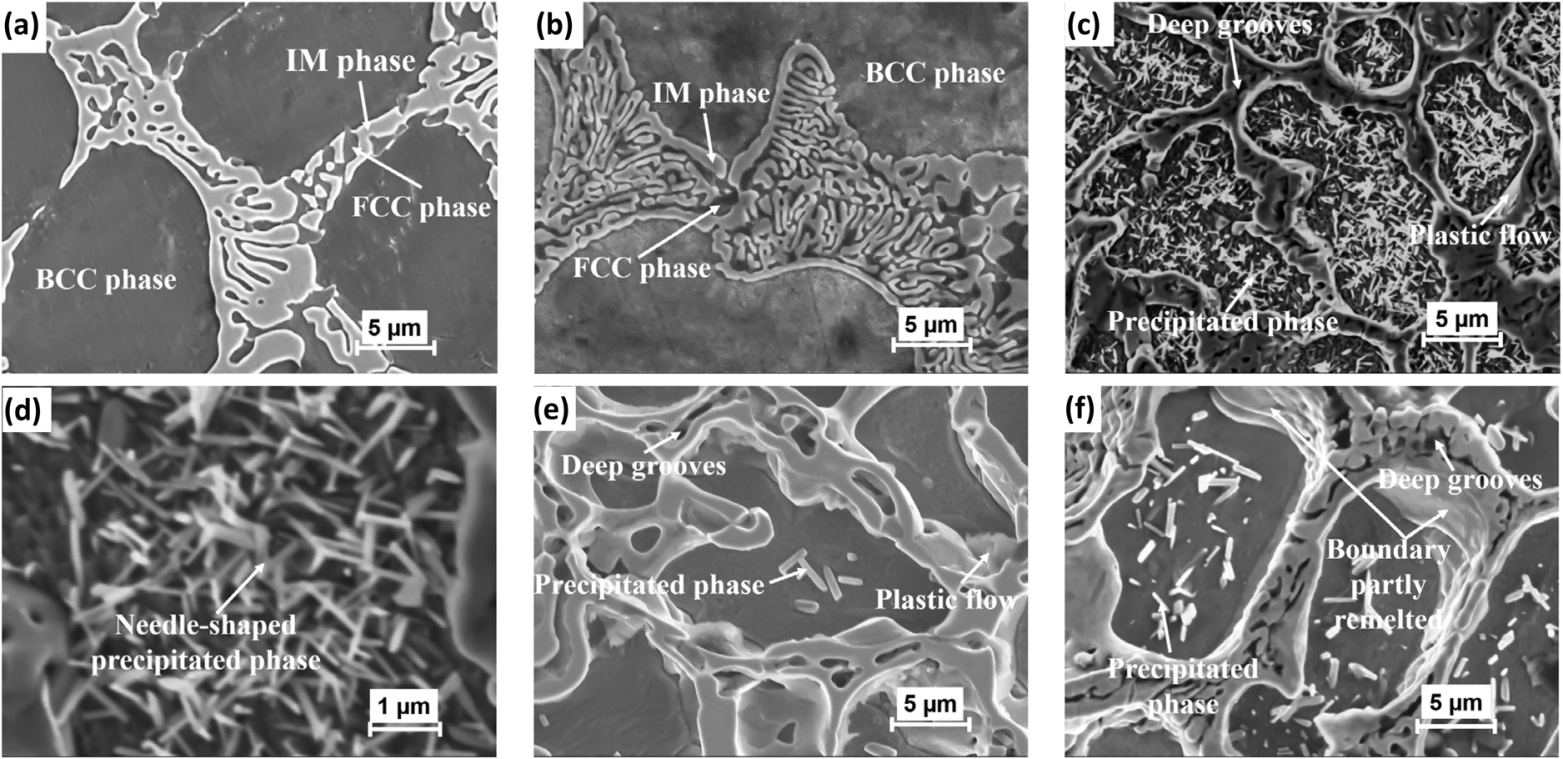
The secondary electrons SEM images of the annealed alloy: (a) HT-600, (b) HT-700, (c and d) HT-825, (e) HT-1000, (f) HT-1050 with holding time of one hour.

Fig. 6 presents the SEM image of the FeCoNiAl_0_._75_Nb_0_._25_ alloy sample after annealing, with optical imaging in Fig. S2 of the ESI† used as a supplementary technique to identify the present phases. Following annealing at 600 °C, the microstructure almost retains the as-cast form, although the dendritic phases become more uniform and equiaxial. The changes become more noticeable after annealing at 700 °C, where the eutectic phase thickens significantly, mainly due to an increase in the number of eutectic plates. This explains the observed increase in diffraction intensity for the IM phase and a corresponding decrease for the BCC phase. Meanwhile, the solid solution region precipitates numerous small, fine IM phases, each about a few hundred nanometers in size, and forms an interwoven needle-like shape (Fig. 6d) after annealing at 825 °C. The number of this phase decrease significantly when increasing the temperature to 1000 °C. Moreover, a shift and decrease in the peak intensity for the IM phase are observed as the annealing temperature increases from 700 to 825 °C (Fig. 2c and d). It is possible that the eutectic plates (IM phase) start to dissolve into the solid solution phases, leading to the morphology of this phase starting to plastic flow at grain boundaries and form deep grooves as shown in Fig. 6e. At higher temperatures (1050 °C), even for a much shorter holding time (one hour), the boundary can be softened, even partly remelted.

**Fig. 7:**
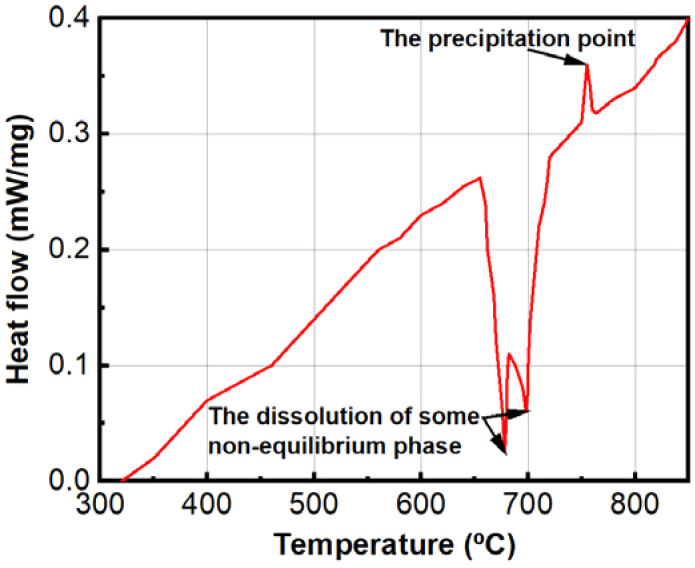
The DSC curve of alloy.

The DSC analysis revealed that phase precipitation commenced at approximately 755 °C (Fig. 7), indicating that needle-shaped precipitated phases are likely to start forming within the BCC phase at this temperature. Additionally, two endothermic peaks were observed around 680–700 °C, which likely correspond to the dissolution of some non-equilibrium phases at these temperatures. This observation could account for the absence of phase precipitation when annealing at 700 °C and the subsequent decomposition of the eutectic phase as previously analyzed.

The EDS analysis results of heat treatment samples are summarized in Table 4. It can be seen that the BCC phase becomes increasingly deplete in Al, and rich in Ni and Co as the annealing temperature increases. That means there has been the diffusion of Al out of the BCC phase formed after casting. In particular, in sample HT-825, the Co and Nb contents increased significantly in this phase. This is likely due to the formation of the Co_2_Nb intermetallic phase, as confirmed by XRD results. The Nb content decreased rapidly with increasing temperature up to 1000 °C along with the decrease in the fraction of the precipitated phase in the BCC phase. The eutectic plate becomes richer in Al, and the remaining phase of the eutectic region maintains an almost constant content when heat-treated to 700 °C. Al tends to diffuse to the grain boundary and thicken this region when the temperature drops below 755 °C, as indicated by DSC data. Above this temperature, the Al and Ni contents rapidly decrease in the eutectic phase. According to the Gibbs phase rule, at higher temperatures, solid solutions are preferentially formed, leading to the dissolution of Al and Ni into the solid solution phase.

### 3.4 Mechanical and magnetic properties

**Fig. 8:**
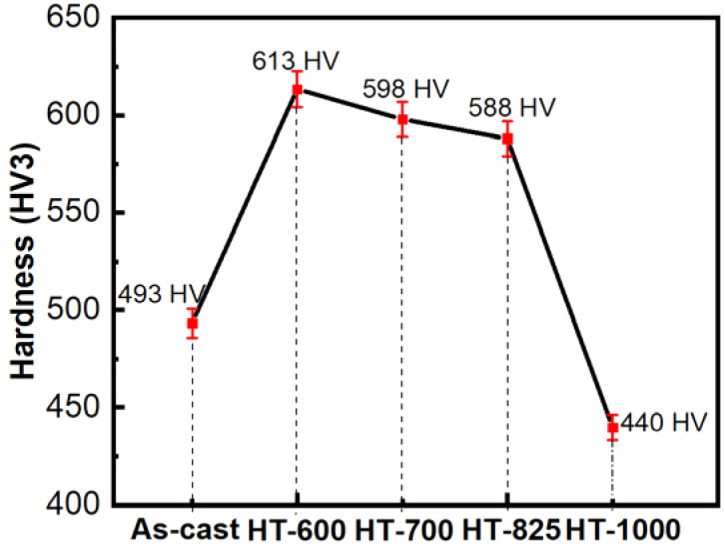
The measured hardness of samples.

As seen above, the microstructure of the alloy consists of a supersaturated solid solution and eutectic phases with a proportion high enough (23%), leading to very high hardness. The tensile specimens of the alloy exhibit brittle behavior, characterized by quite low elongation. Although the ductility of the alloy can be enhanced,^36,37^ however the aim of the alloy is to achieve good magnetic properties while maintaining high strength so ductility is not a focus of this study. The hardness and yield strength of the alloy change significantly with heat treatment. As the annealing temperature approaches 825 °C, both properties increase sharply, stabilizing around 600 HV3 and 2000 MPa, respectively (Fig. 8 and Table 5). This enhancement can be attributed to the thickening of the eutectic phase (a non-equilibrium phase) in conjunction with the precipitation process within the matrix. However, at 825 °C, the decomposition of the eutectic phase initiates, leading to plastic flow at the grain boundaries. As the annealing temperature rises further to 1000 °C, this plastic flow intensifies, resulting in the formation of deep grooves in the microstructure. Consequently, the hardness and yield strength decrease rapidly, reaching 440 HV3 and 1437 MPa, respectively.

**Table 5:** The measured mechanical properties, including Vickers hardness (HV3, MPa) and compressive yield strength (*σ*_*y*_, MPa) of alloy

Alloys	HV, MPa	*σ*_*y*_, MPa	Ref.
FeCoNiAl_0.75_Nb_0.25_ as-cast	4832	1611	This work
FeCoNiCrNb_0.25_ as-cast	2940	423	Jiang *et al.*^25^
AlCrFeNiCu+5 at% Nb as-cast	2695	1110	Malatji *et al.*^23^
(FeCoNiCrMn)_88_Nb_12_ as-cast	—	1010	Qin *et al.*^24^
FeCoNiAl_0.75_Nb_0.25_ HT-600	6007	2002	This work
FeCoNiAl_0.75_Nb_0.25_ HT-700	5860	1953	This work
FeCoNiAlMn HT-700	3558	1186	Yang *et al.*^40^
FeCoNiAl_0.75_Nb_0.25_ HT-825	5762	1921	This work
FeCoNiAlMn HT-800	3066	1022	Yang *et al.*^40^
FeCoNiAl_0.75_Nb_0.25_ HT-1000	4312	1437	This work
FeCoNiAlMn HT-1000	2994	998	Yang *et al.*^40^
FeCoNiCrMn HT-1000	1350	162	Salishchev *et al.*^20^
FeCoNiCrV HT-1000	5870	1435	Salishchev *et al.*^20^

The high hardness value, as shown in Fig. 8, indicates that the alloy exhibits brittle deformation behavior.^22,38^ For high entropy alloys with brittle behavior (containing significant proportions of BCC and IM phases), the yield strength (*σ*_*y*_) and Vickers hardness (HV) are correlated by Tabor's relationship:^39^6
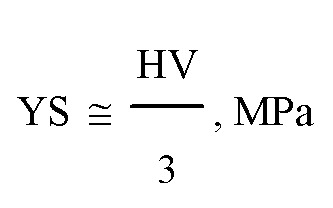


The results of calculations based on this relationship for FeCoNiAl_0.75_Nb_0.25_ alloy are shown in Table 5. The compressive yield strength of the alloy is significantly higher than that of previously published alloys in the as-cast^23–25^ and heat-treated state.^20,40^ The replacement of elements (Cr, Mn, Cu and V) with Al and Nb, and the application of heat treatment significantly increased the hardness and strength of the alloy. The alloy densities calculated using HEAPS software for FeCoNiAl_0.75_Nb_0.25_, FeCoNiCrNb_0.25_, FeCoNiCrMn, FeCoNiCrV, and (FeCoNiCrMn)_88_Nb_12_ are 7.45, 8.23, 8.06, 7.79, and 8.12 g cm^−3^, respectively. The results indicate a reduction in density, which can help decrease the mass of the manufactured product while maintaining high hardness and strength.

**Fig. 9:**
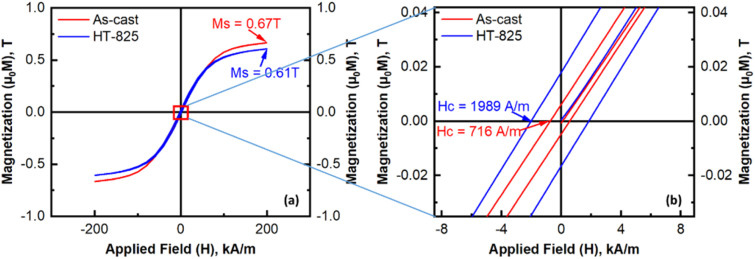
Magnetization hysteresis loops: as-cast and HT-825 specimens.

The precipitation of multiple intermetallic phases in the microstructure did not enhance the magnetic properties of the alloy. In the as-cast state, the values for saturation magnetization (Ms) and coercivity (Hc) were 0.67 T and 716 A m^−1^, respectively. After heat treatment at 825 °C, these values changed to 0.61 *T* for saturation magnetization and 1989 A m^−1^ for coercivity, as shown in Fig. 9. These results indicate that the BCC phase in the as-cast microstructure is ferromagnetic and exhibits good soft magnetic properties. Although the phase at the grain boundary is abundant, up to 23%, it can consist of non-ferromagnetic or weakly ferromagnetic phases. Due to the rapid cooling during casting, Hc remains quite high within the grains. After annealing, even though the amount of precipitated phase increases, Ms decreases only slightly, by about 10%, suggesting the precipitation of weakly ferromagnetic or non-ferromagnetic phases. However, Hc increases significantly because these phases precipitate inside the grains. A comparative analysis with studies on alloys of similar compositions (Table 6) indicates that in the as-cast state, substituting a small amount of Mn with Nb enhances the saturation magnetization (Ms) while maintaining a relatively low coercivity (Hc) compared to the findings of Hariharan *et al.*^41^ However, the Ms values remain considerably lower in both the as-cast and heat-treated states than those reported by Zuo *et al.*,^42^ although the coercivity consistently remains lower. A similar trend is observed for the FeCoNiCu alloy.^43^ This enhancement is slightly superior to that achieved by Cr addition in the FeCoNiAlCr alloy,^22^ and significantly better than the FeCoNiAlCrNb_0.25_ alloy.^22^ Nonetheless, the magnetic properties of the alloy smelted in a vacuum induction furnace are almost equivalent to those of the Mn-containing alloy fabricated using the Laser Metal Deposition (LMD) method.

**Table 6:** Compare magnetic properties of alloys

Alloys	Ms, emu g^−1^ (T)	Hc, Oe (A m^−1^)	Ref.
FeCoNiAl_0.75_Nb_0.25_ as-cast	68.00 (0.67)	9.00 (716)	This work
FeCoNiMnAl_0.7_ as-cast	20.10 (0.19)	13.60 (1082)	Hariharan^41^
FeCoNiMnAl_0.75_ as-cast	100.00 (0.93)	15.00 (1194)	Zuo *et al.*^42^
FeCoNiAlCrNb_0.25_ as-cast	34.69 (0.31)	95.00 (7560)	Ma *et al.*^22^
FeCoNiAlCr as-cast	64.00 (0.57)	52.00 (4138)	Ma *et al.*^22^
FeCoNiAlCu as-cast	84.00 (0.79)	162 (12 892)	Kulkarni^43^
FeCoNiAl_0.65_Mn_0.65_ LMD	78.42 (0.74)	8.57 (699)	Bazioti^44^
FeCoNiAl_0.75_Nb_0.25_ HT-825	62.00 (0.61)	25.00 (1989)	This work
FeCoNiAl_0.75_Mn HT-800	90.00 (0.83)	27.00 (2149)	Zuo *et al.*^42^

In as-cast state, the alloy demonstrates significant hardness and commendable magnetic properties. As a result, it highly suitable for use in magnetic parts that demand superior mechanical properties.

## 4. Conclusions

The influence of microstructure on the mechanical and magnetic properties of the FeCoNiAl_0.75_Nb_0.25_ high-entropy alloy after casting and heat treatment was studied. Changing the heat treatment temperature leads to changes in the phase characteristics of the alloy. The microstructure consists of a mixture of FCC, BCC, and IM phases however their proportions change after heat treatment. The precipitation in the BCC matrix starts at 755 °C and reaches a maximum at 825 °C, then decreases. In the as-cast state, the alloy demonstrates notable magnetic and mechanical properties, with hardness, compressive yield strength, saturation magnetization, and coercive force values of 493 HV, 1611 MPa, 0.67 T, and 716 A m^−1^, respectively. Therefore, it is very suitable for use in magnetic parts requiring superior mechanical properties.

## Data availability

The data supporting this article have been included as part of the ESI.†

## Conflicts of interest

There are no conflicts to declare.

## Supplementary Material

RA-015-D5RA00358J-s001
