# Supplementary material for: The effect of microstructure on mechanical and magnetic properties of FeCoNiAl0.75Nb0.25 high-entropy alloy
Source: RSC Adv. 2025 Mar 6;15(9):7172–80. doi: 10.1039/d5ra00358j (PMC11883541; doi:10.1039/d5ra00358j)
Supplement: RA-015-D5RA00358J-s001 [file RA-015-D5RA00358J-s001.pdf]

Supplementary Information (SI) for RSC Advances.
This journal is © The Royal Society of Chemistry 2025

Supplementary Information (SI)

The effect of microstructure on mechanical and magnetic properties of $FeCoNiAl_{0.75}Nb_{0.25}$ high-entropy alloy

Minh Duc Le,[ab] *Thanh Hung Nguyen,*[b] *Van Duong Nguyen,*[b] *Mai Khanh Pham*[a] *and Hong Hai Nguyen**[a]

[a]*School of Materials Science and Engineering, Hanoi University of Science and Technology, Hanoi 100000, Vietnam.*

[b]*Faculty of Mechanical Engineering, Le Quy Don Technical University, Hanoi 100000, Vietnam.*

To address phase identification, we employed a combination of optical microscopy (OM) imaging, energy-dispersive spectroscopy (EDS), X-ray diffraction (XRD). SE images were used to analyze the morphology of dendritic and inter-dendritic phases, while phase differentiation was supported by additional characterization techniques. First, an OM image of the etched sample (Fig. S1 and Fig. S2) reveals distinct contrast between different regions, providing a initial estimation of the number of phases.

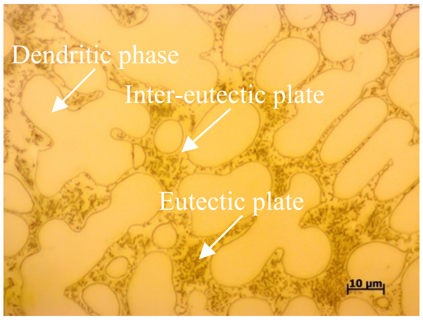

Fig. S1. The microstructure of the as-cast alloy (Optical microscope images)

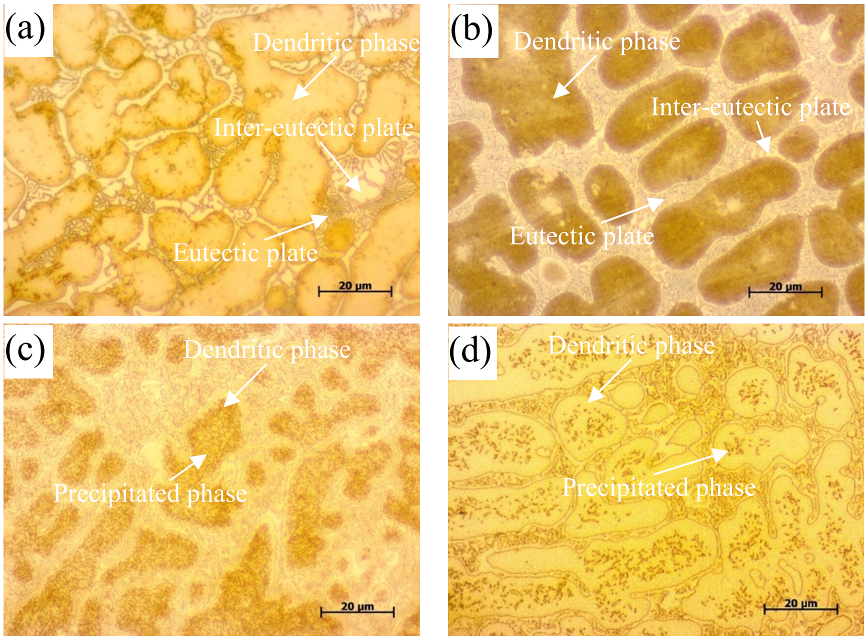

Fig. S2. The microstructure of the alloys (Optical microscope images): (a) HT-600, (b) HT-700, (c) HT-825, and (d) HT-1000.

Next, EDS analysis was performed to determine the composition of these phases, with Fig. S2 showing the locations of EDS analysis for the HT-700 sample. Finally, based on the combined analysis of composition (EDS) and crystal structure (XRD), we identified and labeled the phases.

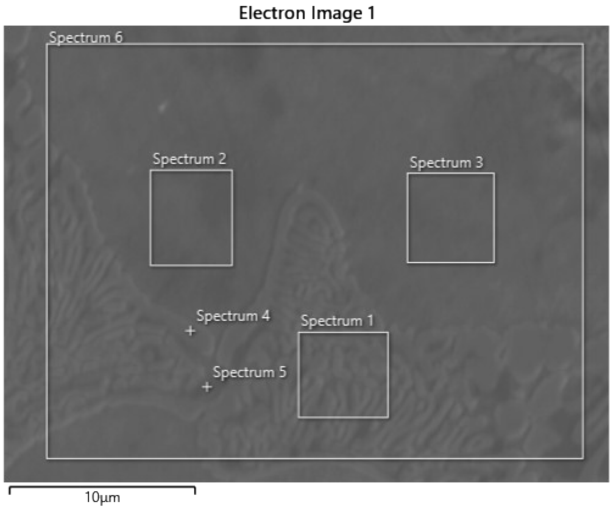

Fig. S3. The location of EDS analysis.

**Spectrum 1**								
Element	Line Type	Apparent Concentration	k Ratio	Wt%	Wt% Sigma	Standard Label	Factory Standard	Standard Calibration Date
Al	K series	0.20	0.00143	3.85	0.42	Al2O3	Yes	
Fe	K series	1.41	0.01408	24.75	2.58	Fe	Yes	
Co	K series	1.46	0.01464	26.66	3.40	Co	Yes	
Ni	K series	1.40	0.01399	24.73	4.63	Ni	Yes	
Nb	L series	0.85	0.00852	20.02	1.91	Nb	Yes	
Total:				100.00				

**Spectrum 2**								
Element	Line Type	Apparent Concentration	k Ratio	Wt%	Wt% Sigma	Standard Label	Factory Standard	Standard Calibration Date
Al	K series	0.49	0.00349	9.97	1.08	Al2O3	Yes	
Fe	K series	1.31	0.01305	23.61	3.13	Fe	Yes	
Co	K series	1.27	0.01270	24.16	4.02	Co	Yes	
Ni	K series	2.00	0.01998	36.97	5.42	Ni	Yes	
Nb	L series	0.21	0.00212	5.29	1.33	Nb	Yes	
Total:				100.00				

Spectrum 3								
Element	Line Type	Apparent Concentration	k Ratio	Wt%	Wt% Sigma	Standard Label	Factory Standard	Standard Calibration Date
Al	K series	0.44	0.00319	9.95	0.82	Al2O3	Yes	
Fe	K series	1.13	0.01131	22.50	2.36	Fe	Yes	
Co	K series	1.28	0.01278	26.66	3.25	Co	Yes	
Ni	K series	1.68	0.01677	34.01	4.14	Ni	Yes	
Nb	L series	0.25	0.00251	6.88	1.07	Nb	Yes	
Total:				100.00				

The chemical composition of the dendritic phase (wt.%) is the average of spectrum 2 and 3. It shows that this phase is enriched in Ni and Al and depleted in Nb and Fe. The nature of such observation corresponds to the BCC phase, as Al and Ni promote the formation of this phase.

The chemical composition of the eutectic phase (wt.%) is spectrum 1. This phase is rich in Fe, Co and Nb, but depleted in Al and Ni.

Spectrum 4								
Element	Line Type	Apparent Concentration	k Ratio	Wt%	Wt% Sigma	Standard Label	Factory Standard	Standard Calibration Date
Al	K series	0.11	0.00082	2.35	0.11	Al2O3	Yes	
Fe	K series	1.22	0.01217	23.40	0.89	Fe	Yes	
Co	K series	1.56	0.01559	30.77	1.20	Co	Yes	
Ni	K series	0.71	0.00714	13.65	1.73	Ni	Yes	
Nb	L series	1.19	0.01188	29.84	0.88	Nb	Yes	
Total:				100.00				

The chemical composition of eutectic plate is spectrum 4. the eutectic plates are rich in Co and Nb, suggesting they likely belong to the (AlNb)Co Laves phase.

Spectrum 5								
Element	Line Type	Apparent Concentration	k Ratio	Wt%	Wt% Sigma	Standard Label	Factory Standard	Standard Calibration Date
Al	K series	0.17	0.00123	3.83	0.22	Al2O3	Yes	
Fe	K series	1.58	0.01582	30.43	1.47	Fe	Yes	
Co	K series	1.47	0.01467	29.49	1.73	Co	Yes	
Ni	K series	1.59	0.01591	31.16	2.31	Ni	Yes	

Nb	L series	0.19	0.00194	5.09	0.51	Nb	Yes	
Total:				100.00				

The chemical composition of inter-eutectic plate is spectrum 5. This phase is rich in Fe, Co, and Ni, but deplete in Al, corresponding to the FCC phase.
